# Parental hesitancy against COVID-19 vaccination for children and associated factors in Taiwan

**DOI:** 10.1186/s12889-023-15158-0

**Published:** 2023-03-27

**Authors:** Jing-Shan Deng, Jau-Yuan Chen, Xiao-Qing Lin, Chun-Lian Huang, Tao-Hsin Tung, Jian-Sheng Zhu

**Affiliations:** 1 Department of Infectious Diseases, Taizhou Hospital of Zhejiang Province Affiliated to Wenzhou Medical University, 150 Ximen Street, Linhai, 317000 Zhejiang Province China; 2 Department of Family Medicine, Chang Gung Memorial Hospital Linkou Branch, Taoyuan City, Taiwan; 3 Evidence-based Medicine Center, Taizhou Hospital of Zhejiang Province Affiliated to Wenzhou Medical University, 150 Ximen Street, Linhai, 317000 Zhejiang Province China

**Keywords:** COVID-19, Vaccine hesitancy, Parents, Children, Taiwan

## Abstract

**Background:**

Since July 2021, some countries and regions have initiated the vaccination of minors against coronavirus disease (COVID-19), and parental COVID-19 vaccine hesitancy will affect the vaccination of minors. We aimed to identify the level of parental hesitancy to vaccinate their children against COVID-19 in Taiwan and the factors associated with vaccine hesitancy.

**Methods:**

We conducted a population-based, self-administered online questionnaire in Taiwan to assess parental hesitancy and the factors influencing their children’s vaccination against COVID-19.

**Results:**

Among 384 respondents, 64.1% were hesitant to have their children vaccinated against COVID-19. Mothers were more likely to hesitate to vaccinate their teens than their fathers (67.5% vs. 50%, *P* < 0.005). Multiple regression results showed that parents who were hesitant to vaccinate themselves (OR = 3.81, 95% CI:2.07–7.02) and those who scored lower on their perception of their children’s vaccination (OR = 9.73, 95% CI:5.62–16.84) were more hesitant to vaccinate their children with COVID-19 vaccine.

**Conclusions:**

According to the study findings, 64.1% of Taiwanese parents were hesitant to vaccinate their children against COVID-19. Parents who were hesitant to receive the COVID-19 vaccine for themselves and had negative views of the vaccine for their children were more likely to be hesitant to vaccinate their children. An in-depth discussion of the factors affecting vaccine hesitancy and targeted health education is conducive to promoting vaccination in children with COVID-19.

## Background

Since December 2019, COVID-19 has spread unexpectedly worldwide and has had a massive effect on people’s fitness and existence [1]. Since the outbreak of the pandemic, a collection of measures, including social distancing, sporting masks, and usual hand washing, have decreased the spreading and mortality of the virus. With the meaningful improvement and examination of a range of COVID-19 vaccines, governments around the world have taken COVID-19 vaccination as an essential approach to resolving the COVID-19 epidemic, so they pay specific attention to the COVID-19 vaccine vaccination work [2]. To contain the pandemic, immunizing the population is essential, and the most reliable way to do this is through mass vaccination [3]. To mitigate the transmission of the new coronavirus, it was estimated that 67% of the population wants to receive a vaccine to acquire the impact of herd immunity [4], and increasing vaccination rates in children is important for herd immunization. A survey conducted in Singapore [5] revealed that children received the COVID-19 vaccine at a substantially slower rate than adults and adolescents, highlighting the significance of raising childhood vaccination rates for herd immunization.

Although nations have had incredible achievements in creating vaccines, the hesitancy of people towards the COVID-19 vaccine is still an important factor that hinders its popularity and coverage of the COVID-19 vaccine [6]. The World Health Organization (WHO) Strategic Advisory Group of Experts on Immunization (SAGE) defines vaccine hesitancy as a “Delay in the acceptance or refusal of vaccination despite the availability of vaccination services” [7]. However, vaccine hesitancy remains widespread because of vaccine development. The hesitation toward vaccines can lead to a decline in vaccine coverage and is not conducive to controlling the spread of epidemics [8].

Children’s bodily features and immune structures have not yet fully developed, and the chance of contracting new coronary pneumonia is higher; therefore, it is necessary for youngsters to be vaccinated against COVID-19 [9]. However, it was discovered that many of the parents interviewed in a study of the factors influencing parental hesitancy to immunize children aged 5–11 in Quebec expressed less concern about the risk of children contracting COVID-19 because they believed there was a low risk of COVID-19 complications [10], which is obviously not conducive to protecting children’s health during a pandemic. The hesitancy of parents to get their young ones vaccinated against COVID-19 is one of the major factors hindering childhood vaccination. The existence of this phenomenon is no longer conducive to achieving herd immunity for the duration of the pandemic [11]. Therefore, it is of great importance to learn about the factors that influence parents’ hesitancy to vaccinate their children against COVID-19 to enhance the vaccination rate of COVID-19 and control the outbreak of COVID-19. The purpose of this study is to investigate the reasons why Taiwanese parents hesitate to vaccinate their children against the novel coronavirus.

## Methods

### Study design and population

We performed a cross-sectional online survey of parents in Taiwan from July 14, 2021, to September 23, 2021. Google was used as the survey platform, and the response rate was 100%. Our target population was parents of at least one child below the age of 18, and 384 questionnaires were collected. Parents who refused to participate in the survey and those without children under the age of 18 living at home were excluded from the study. This study was approved by the Ethics Committee of Taizhou Hospital, Zhejiang Province (approval number: K20210520). All strategies were conducted in accordance with the guidelines of the Institutional Ethics Committee and adhered to the Declaration of Helsinki, and all participant data were anonymized. Ethics Committee of Taizhou Hospital,Zhejiang Province waived informed consent from participants.

### Structured questionnaire

A self-administered questionnaire was designed [12]. The contents of the questionnaire are as follows: (1) basic demographic information, such as gender, region of residence, education level, occupation, and number of minor children; (2) Knowledge about the COVID-19 vaccine: do you know about the COVID-19 vaccine? (Yes, No), do you think the COVID-19 vaccine is effective in preventing the COVID-19 outbreak? (effective, ineffective), how protective do you suppose the COVID-19 vaccine is? (safe, unsafe), and do you assume the COVID-19 vaccine has a preventive impact on COVID-19? How big is it? (Greater effect, lesser effect); (3) Hesitation regarding COVID-19 vaccination for children: Are you hesitant to have your children vaccinated? (Yes; No); (4) Willingness to have teenagers vaccinated against COVID-19?: Score of opinion on children’s vaccination against COVID-19 (Q1: < 30; Q2:≥30, the greater the score, the more advantageous the parents’ view of their child’s vaccination toward the COVID-19.)

### Statistical analysis

The primary finding of the survey was the parental reluctance to vaccinate their children against COVID-19. General demographic features and vaccinations are described using component proportions [n(%)]. To identify the probable causes of parental hesitation to vaccinate their children with the COVID-19 vaccine, a chi-square test was used. The causes of vaccine reluctance were investigated using logistic regression analysis.

Variables significant at *P* < 0.2 level in chi-square or t-test were included in the binary logistic regression model. The factors associated with parental hesitation to vaccinate their children with COVID-19 were determined using binary logistic regression analysis, and the dominance ratio (OR) and 95% confidence interval (CI) were calculated. Statistical significance was considered at *P* < 0.05, using the IBM SPSS statistical software.

### Literature search strategy

We searched the PubMed database for relevant studies published from inception to July 1, 2022. Terms related to key concepts, include vaccine hesitancy, parents, children, and vaccines. Duplicates were removed by scanning the titles and abstracts, resulting in 20 relevant documents. We used a data extraction form to extract the following data from the included studies: first author, study design, study duration, and study duration.

## Results

In this study, 384 participants completed the questionnaire. Table 1 shows that among the 384 parents, 19.8% (76/384) were male, 80.2% (308/384) were female, 15.4% (59/384) lived in township areas, 84.6% (325/294) lived in urban areas, 43.0% (165/384) were employed as medical workers, and 57.0% (219/384) were employed as nonmedical workers.

**Table 1:** Basic characteristics of parents in the study (*n* = 384)

Variables	Categories	Parents (*n* = 384)	Fathers (*n* = 76)	Mothers (*n* = 308)
Residence	Rural/Town	59(15.4)	11(14.5)	50(16.2)
	Urban	325(84.6)	73(96.1)	259(84.1)
Whether the only child	Yes	145(37.8)	32(42.1)	113(36.6)
	NO	239(62.2)	35(59.9)	195(63.3)
Educational level	High school and below	38(9.9)	6(7.9)	32(10.4)
	Bachelor degree or above	346(90.1)	70(92.1)	276(89.6)
Have you received a flu vaccination?	Yes	289(75.3)	52(68.4)	237(76.9)
	No	95(24.7)	24(31.6)	71(23.1)
Whether vaccinated (of any kind)	Yes	60(15.6)	6(7.9)	54(17.5)
	No	224(58.3)	70(92.1)	254(82.5)
a medical worker	Yes	165(43.0)	35(46.1)	129(41.9)
	NO	219(57.0)	41(53.9)	179(58.1)

**Fig. 1:**
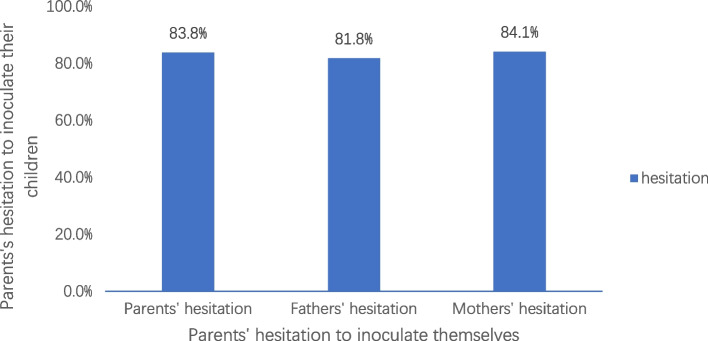
Parents’ hesitancy about their children getting vaccinated against COVID-19

Among the 384 parents, 64.1% (246/384) expressed hesitance to have their children vaccinated against COVID-19, 67.5% (208/308) mothers were hesitant to vaccinate their children against COVID-19, and 50% (38/76) fathers were hesitant to vaccinate their children against COVID-19 (Table 2). Of the 247 parents who were themselves hesitant to take the COVID-19 vaccination, 83.8% (207/247) were also hesitant to have their children vaccinated against COVID-19; Of the 137 parents who were themselves not hesitant to take the COVID-19vaccination, 71.5% (98/137) parents did not hesitate to have their children vaccinated against COVID-19. In addition, of the 247 parents who were hesitant to be vaccinated, 81.8% (6/33) were fathers, and 84.1% (180/214) of mothers were hesitant to have their children vaccinated (Fig. 1).

**Table 2:** Parents’ hesitancy about their children getting vaccinated against COVID-19 (*n* = 384)

	Hesitation (*n* = 246)	No hesitation (*n* = 138)
Parents (*n* = 384)	246(64.1%)	138(35.9%)
Fathers (*n* = 76)	38(50%)	38(50%)
Mothers (*n* = 308)	208(67.5%)	100(32.5%)

Table 3 shows that parents’ hesitation to vaccinate their children with the COVID-19 vaccine was associated with gender (χ2 = 8.097,*p* = 0.004), knowledge of the COVID-19 vaccine (χ2 = 31.17,*p* < 0.001), perception of the safety of COVID-19 vaccine (χ2 = 27.041,*p* < 0.001), perception of the preventive effect of COVID-19 on COVID-19 preventive effect (χ2 = 5.395,*p* = 0.02), perceived effectiveness of COVID-19 vaccine (χ2 = 8.818, *p* = 0.093), and perception score of COVID-19 vaccination for children (t = − 4.081,*p* < 0,001) were related. Moreover, there was a correlation between parents’ hesitation to vaccinate themselves and their hesitation to vaccinate their children (χ2 = 117.217, *P* < 0.001).

**Table 3:** Univariate analysis of factors associated with parents’ hesitancy to have their children vaccinated with the COVID-19 vaccine (*n* = 384)

Variables	Categories	Parents (*n* = 384)		t/χ2	*P*	Fathers (*n* = 76)		t/χ2	*P*	Mothers (*n* = 308)		t/χ2	*P*
		Hesitation (*n* = 246)	No Hesitation (*n* = 138)			Hesitation (*n* = 38)	No Hesitation (*n* = 38)			Hesitation (*n* = 208)	No Hesitation (*n* = 100)		
Sex	Male	38(50)	38(50)	8.097	0.004	38(50)	38(50)			/	/		
	Female	208(84.6)	100(26.7)			/	/			208(84.6)	100(26.7)		
Residence	Urban	205(83.3)	120(32.1)	0.892	0.345	34(44.7)	31(40.8)	0.957	0.328	171(55.5)	89(28.9)	2.366	0.124
	Rural/Town	41(16.7)	18(4.8)			4(5.3)	7(9.2)			37(12.0)	11(3.6)		
Educational level	High school and below	24(6.3)	14(3.6)	0.015	0.903	3(3.9)	3(3.9)	0.000	1.000	21(5.5)	11(3.6)	0.059	0.808
	Bachelor degree or above	222(57.8)	124(32.3)			35(46.1)	35(46.1)			187(60.7)	189(61.4)		
Whether is a medical worker	Yes	108(28.1)	57(14.8)	0.244	0.622	22(28.9)	13(17.1)	1.774	0.183	86(27.9)	44(14.3)	0.001	0.980
	No	138(35.9)	81(21.1)			20(26.3)	21(27.6)			118(38.3)	60(19.5)		
one-child family	Yes	95(24.7)	50(13.0)	0.214	0.644	17(22.4)	15(19.7)	0.216	0.642	78(25.3)	35(11.4)	0.182	0.670
	No	151(39.3)	88(23.9)			21(27.6)	23(30.3)			130(42.2)	65(21.1)		
Knowledge of COVID-19	Yes	114(29.7)	85(22.1)	8.238	0.004	22(28.9)	28(36.8)	2.921	0.087	91(29.5)	53(17.1)	3.747	0.053
	NO	185(48.2)	53(13.8)			16(21.1)	9(11.8)			113(36.7)	42(13.6)		
Prevention against Covid-19	Great effect	169(44.0)	110(28.6)	5.395	0.020	23(30.3)	35(46.1)	10.483	0.001	146(47.4)	75(24.4)	0.770	0.380
	Small effect	77(20.1)	28(7.3)			15(19.7)	3(3.9)			62(20.1)	25(8.1)		
Effectiveness in prevention COVID-19	Effective	187(48.7)	115(29.9)	8.818	0.093	31(8.1)	34(44.7)	0.957	0.328	156(50.6)	81(26.3)	1.371	0.242
	Not effective	59(15.4)	23(6.0)			7(9.2)	4(5.3)			52(16.9)	19(6.2)		
Hesitancy of parents to receive vaccines against COVID-19	Yes	207(53.9)	40(10.4)	117.217	<0.001	27(35.5)	6(7.9)	23.619	<0.001	180(58.4)	34(11.0)	87.908	<0.001
	No	39(10.2)	98(25.5)			11(14.5)	32(42.1)			28(9.1)	66(21.4)		
Score of knowledge about COVID-19													
<30		136(35.4)	24(6.3)	52.228	<0.001	27.4 ± 6.7	33.2 ± 5.6	−4.081	<0.001	26.7 ± 5.2	32.7 ± 4.0	−7.097	<0.001
≥ 30		110(28.6)	114(29.7)										
Safety of COVID-19 vaccines	Safe	94(24.5)	94(24.5)	31.638	<0.001	14(18.4)	25(32.9)	9.988	0.002	78(25.3)	60(19.5)	16.289	<0.001
	Unsafe	152(39.5)	44(11.5)			21(27.6)	7(9.2)			129(41.9)	36(11.7)		

Among them, perception of the safety of the COVID-19 vaccine, knowledge of the COVID-19 vaccine, and score of perception of children’s vaccination against new crowns were associated with fathers’ and mothers’ hesitation to vaccinate their children against the COVID-19 vaccine (*P* < 0.2). In addition, fathers’ hesitation to vaccinate their children with the COVID-19 vaccine was associated with whether their occupation was medical (χ2 = 1.774, *P* = 0.183) and their perception of the preventive effect of COVID-19 vaccine on COVID-19(χ2 = 10.483, *P* = 0.001), whereas mothers’ hesitation to vaccinate their children with COVID-19 vaccine was associated with the type of residence (χ2 = 2.366, *P* = 0.124).

The results of the regression model are presented in Table 4. Parents who were hesitant to vaccinate themselves with the new crown vaccine (OR = 3.81, 95% CI:2.07–7.02) and those who scored lower on their perceptions of their children’s COVID-19 vaccination (OR = 9.73, 95% CI:5.62–16.84) were more hesitant to vaccinate their children with the COVID-19 vaccine. Parents’ type of residence, education, whether they were an only child, and whether they were medical workers were not significant in the multiple regression model and thus can be considered confounding factors.

**Table 4:** Multiple logistic regression analysis of factors associated with parents’ hesitancy to have their children vaccinated with the COVID-19 vaccine (*n* = 384)

Variables	Categories	Parents		Fathers		Mothers	
		*P*	OR	*P*	OR	*P*	OR
Sex	Female vs. male	0.543	0.81(0.43–1.55)				
Residence	Rural town vs. urban					0.010	0.30(0.12–0.75)
Educational level	High school and below Bachelor’s degree or above						
a medical worker	Yes vs. No			0.523	1.55(0.40–5.98)		
Knowledge of Covid-19	Understanding vs. don’t know	0.855	0.95(0.56–1.61)	0.671	0.76(0.21–2.66)	0.883	0.95(0.52–1.73)
Prevention against Covid-19	Great vs. small	0.835	1.07(0.54–2.10)	0.268	0.39(0.07–2.04)		
Effectiveness in prevention Covid-19	Effective vs.no effective	0.637	1.18 (0.58–2.37)				
Hesitancy of parents to receive vaccines against COVID-19	Yes vs. No	<0.001	3.81(2.07–7.02)	0.001	12.27(2.80–53.61)	<0.001	10.92(5.80–20.58)
Score of knowledge about Covid-19	Q1vs.Q2	<0.001	9.73(5.62–16.84)	0.010	12.27(2.80–53.61)	0.001	3.19(1.60–6.34)
Safety of Covid-19 vaccines	Safe vs. unsafe	0.415	0.78(0.43–1.40)	0.901	0.91(0.20–4.04)	0.412	0.76(0.40–1.44)

Both fathers’ and mothers’ hesitation to vaccinate their children with the COVID-19 vaccine were associated with scores on perceptions of their children’s vaccination and hesitation to vaccinate themselves with the COVID-19 vaccine. The type of residence was associated with mothers’ hesitation to vaccinate their children against COVID-19 (OR = 0.30,95% CI:2.07–7.02), whereas fathers were not affected by this factor.

## Discussion

### Impact of vaccine hesitancy

Vaccine hesitancy is a phenomenon that has always existed and has caused great harm to people’s health [ 13]. Although a COVID-19 vaccine has been developed, vaccine hesitancy is not conducive to the global control of the current COVID-19 pandemic, and the existence of this phenomenon will have adverse effects on people’s physical health, mental health, and social economy [6].

According to the results of this survey, 64.1% (246/384) of the parents were hesitant to have their children vaccinated against COVID-19. The hesitation of parents to vaccinate their children against COVID-19 in the various study populations is listed in Table 5. These studies emphasize the importance of community immunity as a means of containing the COVID-19 pandemic, and most of them have discovered that parents’ hesitation in having their children vaccinated against COVID-19 has a minimal bearing on vaccine safety [10, 11, 14–32]. Parents in various countries or areas are typically hesitant to have their children vaccinated against COVID-19. According to an analysis of the hesitancy of parents in those countries or regions to have their children vaccinated, hesitation rates range from 8 to 88%.

**Table 5:** Parental vaccination of their children against COVID-19 in different study populations

Author	Study design	Study period	Sample size	Setting	Hesitancy (%)	Reference
Evangelista Bagateli et al	cross-sectional study	2021	Total: 501	Brazil	Total:8%	[14]
Yulia Gendler et al	cross-sectional study	2021	Total: 520	Israeli	Total:29.6%	[15]
Eve Dubé et al	cross-sectional study	2021	Total: 28	Canada	Total:75%	[10]
Amornphat Kitro et al	cross-sectional study	2021	Total: 1064	Thailand	Below 12 years:56.9%; Over 12 years:17.1%	[16]
Grazia Miraglia del Giudice et al	cross-sectional study	2021–2022	Total: 430	Italy	Total: 37%	[17]
Ting Li et al	cross-sectional study	2021	Total: 3484	China	Total: 20.7%	[18]
Chia-shi Wang et al	cross-sectional study	2020–2021	Total: 207	US	Total: 61%	[19]
Sawsan Abuhammad et al	cross-sectional study	2021	Total: 1078	Jordan	Total: 77.6%	[20]
Gabriellla Di Giuseppe M.D. et al	cross-sectional study	2021	Total: 607	Italy	Total: 31.5%	[21]
Mohamad-Haani-Temsah	cross-sectional study	2021	Total: 3167	Saudi Arabia.	Total:52.4%	[22]
Qiang Wang	cross-sectional study	2020	Total: 3009	WuXi	Total:40.17%	[23]
Alfieri, Nian. L	cross-sectional study	2020	Total: 1425	Chicago	Total:67%	[24]
Micah A. Skeens	cross-sectional study	2021	Total: 491	US	Total:18.5%	[25]
Chloe A. TeasdalePhD	cross-sectional study	2021	Total: 2506	US	Total:88.1%	[26]
Sarah Musa	cross-sectional study	2020	Total: 4203	Qatar	Total:17.9%	[27]
Amy B. Middleman	cross-sectional study	2020	Total: 900	US	Toatl:26%	[28]
Huynh, G.	cross-sectional study	2021	Total: 1015	Vietnam	Total:26.2%	[29]
Samantha Schilling	cross-sectional study	2021	Total: 50	US	Total:62%	[30]
Marco Montalti	cross-sectional study	2021	Total: 4993	Italy	Total:9.9%	[31]
Yunyun Xu	cross-sectional study	2021	Total: 917	China	Shangdong:19.4% Zhejiang:111.7%	[32]
Wilfred Hing-sang Wong	cross-sectional study	2022	Total: 545	China	at the beginning of the seminar:40% at the end of the seminar:66%	[11]

### Influencing factors of vaccine hesitancy

Our study showed a correlation between parents’ hesitation to vaccinate themselves and their hesitation to vaccinate their children, with parents who were more hesitant to vaccinate themselves being more hesitant to vaccinate their children. A study of parental hesitancy to vaccinate children against COVID-19 in the United States [11] showed a significant correlation between parents’ intention to vaccinate against COVID-19 and their intention to vaccinate their children against COVID-19. This provides new ways to improve COVID-19 vaccine coverage in children. Studies have shown that promoting the health of children is an important reason why parents are willing to vaccinate their children against COVID-19 [33]. Therefore, there is a need to raise awareness among parents about the importance of COVID-19 vaccination, to understand the important role of the COVID-19 vaccine for children’s health during a pandemic, to give parents correct information about the COVID-19 vaccine, and to increase acceptance and trust of the vaccine among adults and, to some extent, children’s vaccination rates.

Vaccine hesitation is a complex social phenomenon influenced by environmental, social, cultural, and political factors in addition to individual attitudes and beliefs [34]. A study examining the growing trend of parental vaccine hesitancy suggested that many factors influence parental hesitancy about vaccines for their children, divided into parent-specific factors (race, income, education level, knowledge of vaccines, past experiences), vaccine-specific factors (perceived vaccine effectiveness, perceived vaccine safety, perceived disease susceptibility), and external factors (policy, media, social norms, school immunization requirements, etc.) [35]. Our survey results showed that the lower the perception score of their child’s COVID-19 vaccination, the more hesitant parents were to vaccinate their children. This is sufficient to show that parental attitudes regarding COVID-19 vaccination can, to some extent, influence their decisions to vaccinate their children against COVID-19. In a cross-sectional study [15] investigating the effects of vaccine literacy, vaccine perceptions, and vaccine hesitancy on Israeli parents’ willingness to get COVID-19 vaccine for their children, vaccine literacy, perceived vaccine hesitancy, and COVID-19 vaccine perceptions were found to be associated with parents’ willingness to vaccinate their children against COVID-19. This is similar to the results of our study, which demonstrated that parental attitudes, perceptions of the COVID-19 vaccine, and literacy are influential factors in parents’ willingness to vaccinate their children against COVID-19.

In line with the results of other studies [36–38], in the current study, we found that women living in rural areas were more hesitant to have their children vaccinated with COVID-19 than those living in urban areas. In a study on attitudes toward the COVID-19 vaccine and vaccine hesitancy among rural and urban communities in Tamil Nadu, India, it was found that residents living in urban areas had more trust in the effectiveness of the vaccine and that they did not prefer natural immunization, while women from rural areas had less trust in the health system, COVID-19 vaccine, and its effectiveness [39]. In addition, in a study of hesitant adults in Arkansas who had received the COVID- 19 vaccine, it was found that people living in rural areas were more hesitant to be vaccinated, possibly because of the high cost of vaccination and the low rates of influenza vaccination in the past few years [40]. These results suggest that we need to increase awareness of vaccines in rural areas, improve healthcare systems in rural areas, provide more medical resources in rural areas, and proactively address the health and social inequalities created during pandemics.

Concerns regarding vaccine safety have long been one of the most common reasons for vaccination. Multiple studies have found that those who are hesitant to get the COVID-19 vaccine or to vaccinate their children cite safety as a primary concern [28]. In mainland China, although the vaccination rate for the new crown vaccine is high, many respondents still expressed concerns about the safety and side effects of the vaccine [41]. Studies have shown that healthcare providers and the Internet are the most common sources of information on COVID-19 vaccines [15]. Therefore, government departments need to actively cooperate with the healthcare sector to take advantage of the rapidity and extensiveness of the Internet in information dissemination to convey information about the safety and efficacy of the COVID-19 vaccine to the general public and to enhance public understanding and information about the vaccine.

### Limitations

Although a population-based study was conducted, this study has the following limitations. First, there were limitations in the selection of research objects. The samples in this survey were unevenly distributed in terms of sex and educational level. Second, this is a cross-sectional study, which can only reflect the situation at a specific point in time and cannot reflect the changes in people’s attitudes at different times. Finally, the questions on the questionnaire were limited and could not reflect other influencing factors such as differences in policies and cultures between Taiwan and other regions. These factors require further investigation.

## Conclusion

Vaccine hesitancy is an important public health issue with multiple root causes. According to the study’s findings, 64.1% of Taiwanese parents were hesitant to vaccinate their children against COVID-19. Parents who were hesitant to get the COVID-19 vaccine for themselves and had negative views of the vaccine for their children were more likely to be hesitant to vaccinate their children. Promoting the vaccination of children with the COVID-19 vaccine requires a thorough examination of the variables that influence vaccine hesitancy, as well as focused health education. Taiwan’s culture, politics, and economics are special, necessitating thorough discussion.

## Data Availability

The datasets used and/or analysed during the current study are available from the corresponding author on reasonable request.
